# Research on Division of Labor Decision and System Stability of Swarm Robots Based on Mutual Information

**DOI:** 10.3390/s24155029

**Published:** 2024-08-03

**Authors:** Zhongyuan Feng, Yi Sun

**Affiliations:** School of Communication and Information Engineering, Xi’an University of Science and Technology, Xi’an 710054, China; fengzhongyuan@stu.xust.edu.cn

**Keywords:** chaotic dynamics, game theory, information theory, mutual information, swarm robotics

## Abstract

In rational decision-making processes, the information interaction among individual robots is a critical factor influencing system stability. We establish a game-theoretic model based on mutual information to address division of labor decision-making and stability issues arising from differential information interaction among swarm robots. Firstly, a mutual information model is employed to measure the information interaction among robots and analyze its influence on the behavior of individual robots. Secondly, employing the Cournot model and the Stackelberg model, we model the diverse decision-making behaviors of swarm robots influenced by discrepancies in mutual information. The intricate decision dynamics exhibited by the system under the disparity mutual information values during the game process, along with the stability of Nash equilibrium points, are analyzed. Finally, dynamic complexity simulations of the game models are simulated under the disparity mutual information values: (1) When *ν*_1_ of the game model varies within a certain range, the Nash equilibrium point loses stability and enters a chaotic state. (2) As *I*(*X*;*Y*) increases, the decision-making pattern of robots transitions gradually from the Cournot game to the Stackelberg game. Concurrently, the sensitivity of swarm robotics systems to changes in decision parameter decreases, reducing the likelihood of the system entering a chaotic state.

## 1. Introduction

The complexity and uncertainty observed in biological populations in nature, such as flocks of birds, schools of fish, and colonies of insects, serve as significant sources of inspiration for research in collective intelligence [1,2,3]. These populations achieve self-organization, cooperation, and adaptation to the environment through simple information exchange among individuals. For instance, during migration, bird flocks dynamically adjust their flight formations based on interactions and information sharing among individuals to cope with diverse environmental challenges [4]. Insect colonies, particularly social insects like ants and bees, efficiently search for resources and construct nests through individual autonomous decision-making and information exchange, ensuring the survival and propagation of the entire population [5]. These natural phenomena illustrate the marvels of collective behavior while also highlighting the importance of information exchange in maintaining population stability and facilitating complex decision-making.

Swarm robotics, as an exemplary embodiment of collective intelligence, holds research significance and application prospects in modern technology domains. Individual robots can accomplish complex tasks and decision-making processes through simple rules and local interactions without central control [6,7,8]. To enable swarm robots to effectively accomplish complex tasks such as consensus [9], task allocation [10], synchronization [11], controllability [12], etc., information becomes indispensable. Information interaction not only determines the level of collaboration among individuals within the system but also influences the stability and behavioral characteristics of the entire system [13,14].

In information theory, Shannon quantified the information contained in one random variable about another random variable through mutual information. This concept can be likened to the degree of information interaction between robots [15,16,17]. Parunak et al. first proposed utilizing the concept of mutual information in multi-agent systems to measure correlation, allowing for the definition of coherent, collaborative, cooperative, competitive, and coordinated concepts [18]. Therefore, investigating the influence of information interaction among group robots on decision-making behaviors, particularly studying its impact on overall behavioral stability, represents a worthwhile topic for in-depth exploration.

However, as the complexity of information interaction increases, swarm robotics systems often exhibit astonishing phenomena of emergent chaos. One of the characteristics of chaos theory is its high sensitivity to initial conditions, implying that even small parameter changes in the decision-making of swarm robotics can lead to significant differences in system behavior, a phenomenon commonly referred to as the “butterfly effect” [19,20,21]. In swarm robotic systems, minor variations in information interaction can lead to unpredictability and nonlinear responses in overall system behavior, posing significant challenges for studying system stability.

In summary, investigating the impact of mutual information on the emergence of chaotic phenomena in the decision-making process of swarm robotics systems holds profound significance for analyzing system stability. Specifically, this paper contributes as follows:

1. A mutual information model is built to measure the disparity of information interaction within swarm robot systems. The exponential function is leveraged to convert the mutual information value into a probability. Leveraging this probability value, the assessment of information interaction among diverse robots is conducted to unveil the strategic choices made by individual robots across the disparity of mutual information.
2. The division of labor decision-making behavior of swarm robots under different mutual information levels is modeled as a game-theoretic model, exploring the complex dynamical behaviors exhibited by robots during the game process. Based on this, the stability and bifurcation characteristics of the established dynamic game model are analyzed. The impact of system parameter on the dynamic behavior of the model is investigated, leading to the determination of equilibrium stability conditions and complex features.
3. The conclusions derived and simulated are as follows: ① When the decision parameter $v_{1}$ of the game model varies within a certain range, the Nash equilibrium point loses stability and enters a chaotic state. ② As the level of information exchange between robots increases (i.e., $I\left(X;Y\right)$ increases), the decision-making pattern of robots transitions gradually from the Cournot game to the Stackelberg game. Concurrently, the sensitivity of swarm robotics systems to changes in decision parameter decreases, reducing the likelihood of the system entering a chaotic state.

The structure of the remaining sections of this paper is as follows: Section 2 outlines the relevant studies in game theory, information theory and chaos theory. Section 3 presents the business model, mutual information model and game-theoretic model of swarm robotics systems. Section 4 conducts a stability analysis of the game-theoretic model. Section 5 discusses the simulation results. Finally, Section 6 draws conclusions and outlines future work.

## 2. Related Work

### 2.1. Research Related to Division of Labor and Decision-Making in Swarm Robotic Systems

The division of labor in a swarm robotics system is a complex game-theoretic problem involving multi-party decision-making. Modeling this process using game-theoretic epistemological methods stands as a mainstream research approach. Most researchers align this problem with an economic model, offering theoretical support for analyzing robot behavior and decision-making.

In the field of economics, classical game theory finds extensive application in analyzing the decision-making problems associated with competition and cooperation among markets. Likewise, the problem of the division of labor for a swarm robotics system can be likened to an economic problem, wherein the quantity competition game serves as a common tool for analyzing resource allocation decisions. Du, H., et al. employed importance measure methods to model the mission reliability of UAV swarms and optimize their structure. The research focuses on methodologies aimed at enhancing task reliability and optimizing system structure [22]. Han, S., et al. introduced a modified genetic algorithm for addressing task assignment issues in heterogeneous UAV systems. It integrates game theory models such as Cournot and Stackelberg to improve task allocation efficiency and overall system performance [23]. He Z., et al. discussed the application of the Cournot game theory model for optimizing task allocation in multi-robot systems, with an emphasis on enhancing collaborative efficiency and optimizing decision-making processes within the systems [24].

While the aforementioned research advancements have made significant progress in addressing task allocation issues in swarm robotics systems, the intelligent behaviors and decision-making abilities exhibited by individual robots during actual collaborative processes are achieved through interactions and information sharing among individuals within the group. Therefore, achieving effective collaboration necessitates a deeper investigation into the influence of information exchange among swarm robots on behavioral characteristics.

Yamamoto S., et al. proposed an interactive learning and decision-making method based on reinforcement learning and deep learning for individual robots in multi-agent systems. This study explores how individual robots can enhance their decision-making efficiency in complex environments through information sharing and interactive learning, providing new theoretical perspectives on individual intelligence in group robot systems [25]. Hu F., et al. investigated the use of information-theoretic approaches to optimize collaborative decision-making in group robot systems. The research analyzed the importance of information sharing in enhancing overall system efficiency and coordination, and proposed a decision-making model based on mutual information to achieve effective robot collaboration [26]. Bonnet F., et al. provided a comprehensive review of the adaptive decision-making mechanisms in swarm robotics. The paper discussed how individual robots achieve collective intelligence through adaptation and interaction, and explored the potential of various decision-making algorithms in addressing complex environments and task requirements [27].

The aforementioned literature offers valuable insights for this paper’s investigation into the influence of information interactions among individuals on robot decision-making within the realm of swarm robotics. The interdisciplinarity of information theory and decision theory provides this paper with a novel perspective, contributing to a more profound comprehension of the role played by mutual information in decision-making within swarm robotics.

### 2.2. Research Related to the Stability of Swarm Robotic Systems

The stability of swarm robot systems is a crucial aspect for ensuring their reliable operation and coordination in various applications. Recent studies have increasingly focused on applying chaos theory and control methods to address stability issues within these systems. Tomaselli C., et al. explored the application of chaos synchronization control methods in multi-robot systems, focusing on stability issues arising from nonlinear dynamics. By employing chaos theory, the research presents an effective approach to achieving synchronization among system components, thereby enhancing overall system stability and responsiveness [28]. Ahmadi Balootaki M., et al. proposed novel chaos control methods to stabilize multi-robot systems. Through mathematical modeling and experimental validation, the study demonstrates how control techniques derived from chaos theory effectively suppress potential instability within the system, thereby improving its stability and robustness [29]. Li R., et al. introduced an event-triggered chaos control method designed to address stability issues in multi-agent systems with communication delays. The study shows that this approach significantly reduces communication resource consumption while maintaining system stability and coherence [30].

These studies provide significant insights into the potential for understanding and applying chaos theory to the stability of swarm robotic systems. It is noteworthy that the aforementioned studies did not analyze the causes of chaos phenomena in swarm robotics systems from the perspective of information theory. Hence, to comprehend and analyze chaos phenomena in swarm robotics systems, it is essential to delve into the influence of information interaction on system decision-making behavior.

Hussain A., et al. explored how information transmission through event-triggered control induces chaos synchronization in multi-agent systems. The study found that information interaction has a significant impact on system stability and behavior, providing new insights into the dynamics of complex systems [31]. Wang Z., et al. investigated the event-triggered consensus problem in multi-agent systems with information transmission delays. The results emphasized the impact of information interaction delays on system stability and the achievement of consensus, providing a theoretical foundation for optimizing control strategies [32]. Liu M., et al. analyzed the impact of information exchange on the synchronization performance of multi-robot systems, summarizing the variations in system stability and performance under different information interaction modes [33].

These studies provide in-depth theoretical analyses and empirical research on how information interaction affects the stability of swarm robotic systems. However, they have not analyzed the impact of mutual information between robots on the stability of swarm robotic systems from an economic perspective. Additionally, there is a lack of in-depth research on how changes in the degree of information interaction influence individual robot decision-making behavior, leading to changes in the game-theoretic strategies employed.

## 3. Model

For a generalized model, where the swarm robot system is represented by the set $V=\left\{1,2,\cdots ,V\right\}$. For each $robot_{i}\in V$, define as follows:

(1) $$Robot_{i}=\left(S_{i},B_{i},N_{i},U_{i},I_{i}\right)$$

where

- “$S_{i}$ (State)” signifies information pertaining to the current condition of the robot, encompassing aspects such as strategy, position, etc.
- “$B_{i}$ (Behavior)” embodies the robot’s behavior or decision-making, governing its adaptations and adjustments.
- “$N_{i}$ (Network)” is defined as a complete graph that delineates the information links existing between a robot and its neighboring counterparts.
- “$U_{i}$ (Utility)” describes the utility of the robot, and each individual robot adapts its strategies aiming to maximize utility.
- “$I_{i}$ (Information)” represents the mutual information among robots, reflecting the disparity of information interaction and sharing among them.

### 3.1. Network Topology

This study addresses the issue of the division of labor among swarm robots within complex systems, theoretically examining the impact of varying degrees of information exchange among individual robots on system stability. In the process of collaborative task execution facilitated by the division of labor, decision-making behaviors among individual robots often induce resource flows. Consequently, robots, when making decisions, are influenced not only by interactions with other robots but also by a rational consideration of factors such as energy, losses, and behavioral costs to maximize their own interests. However, such behavior may lead to a deviation of collective interests from the optimal societal utility, as each individual robot, driven by self-interest, engenders competition with others, thereby affecting the overall stability of the system. Using the quantity competition game model, this competitive behavior can be mathematically modeled and the impact of robot strategies on the overall stability of the system can be analyzed under varying degrees of information exchange.

**Figure 1:**
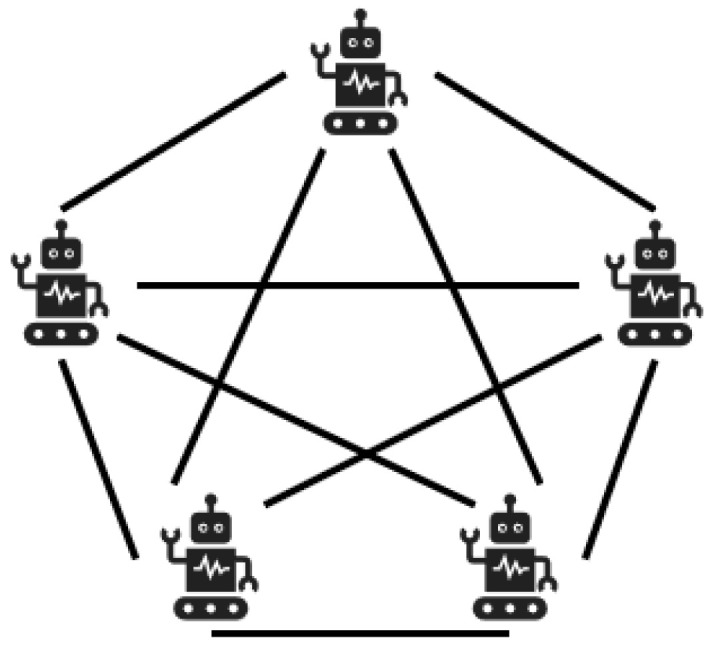
Diagram of swarm robot network topology.

This paper defines the information connectivity among swarm robots as a complete graph, ensuring each robot can directly communicate with every other robot. Even if some communication links fail in dynamic environments, robots can still interact through alternative paths. As illustrated in Figure 1, nodes represent the robots participating in the game, and the edges between nodes represent the strategic interactions among the robots. From Figure 1, it can be seen that in the swarm robot system, there is a game-based relationship between all robots. This means that the behavior and strategy choices of each robot will be influenced by other robots, and their decision-making relationships with each other will generate complex dynamics in the whole system.

### 3.2. Mutual Information Model

The information theory decision methodology represents a decision analysis approach grounded in the fundamental principles of information theory. This methodology holds significant importance, particularly in facilitating research that involves multi-disciplinary cross-pollination. Mutual information, an essential concept derived from information theory, is widely used to measure the extent of correlation and interdependence among various decision variables. If knowledge of Y reduces our uncertainty of X, then we say Y carries information about X. Hence, mutual information serves as a metric to quantify the information interaction among robots throughout the course of the game.

In a swarm robotics system, each resource-providing robot is considered a random variable, while its decision-making $x_{i}$ are regarded as observations within the system. Let X represent a random variable. The information entropy is defined as:

(2) $$H\left(X\right)=-\sum_{i=1}^{N}p\left(x_{i}\right)\log_{2}p\left(x_{i}\right)$$

$H\left(X\right)$ is also recognized as the marginal entropy of X, since it solely relies on the marginal probability distribution of a random variable. After establishing the concept of marginal entropy for a single random variable, this definition can be readily extended to encapsulate the joint entropy of two random variables:

(3) $$H\left(XY\right)=-\sum_{i=1}^{N}\sum_{j=1}^{M}p\left(x_{i},y_{j}\right)\log_{2}p\left(x_{i}y_{j}\right)$$

as well as the conditional entropy of these two random variables:

(4) $$H\left(X\left|Y\right.\right)=-\sum_{i=1}^{N}\sum_{j=1}^{M}p\left(x_{i},y_{j}\right)\log_{2}p\left(x_{i}\left|y_{j}\right.\right)$$

where $p\left(x,y\right)$ denotes the joint probability distribution function, while $p\left(x,y\right)$ represents the marginal probability of Y and X. The mutual information, denoted by $I\left(X;Y\right)$, is defined as:

(5) $$I\left(X;Y\right)=-\sum_{i=1}^{N}\sum_{j=1}^{M}p\left(x_{i},y_{j}\right)\log_{2}\left(\frac{p\left(x_{i},y_{j}\right)}{p\left(x_{i}\right)p\left(y_{j}\right)}\right)$$

When $I\left(X;Y\right)=0$, it indicates a lack of shared information between robot X and robot Y, i.e., there are fewer connections among robots. This scenario might suggest restricted awareness regarding the behavior of other robots or an incapacity to accurately predict the opponent’s strategy. Consequently, the resource-providing robot may display a stronger propensity for independent decision-making. Such a choice exemplifies the characteristics of a Cournot game. In Cournot games, each robot selects its strategy relatively independently, without taking into account the reactions of other robots. Conversely, if $I\left(X;Y\right)>0$, it signifies a higher degree of information exchange and correlation between X and Y, i.e., there are stronger connections among robots. This condition implies that their decision-making might mutually influence one another, suggesting a heightened capacity to comprehend the behavioral patterns or predict the strategies of other robots more accurately. In such instances, the resource-providing robot may lean more towards making decisions based on the information from others. This choice makes the dynamics of the game among swarm robots into characteristics of a Stackelberg game. In a Stackelberg game, the robot possessing superior information assumes the role of the leader, impacting the behaviors of the other robots. The leader robot leverages its informational advantage to formulate optimal strategies and anticipate the responses of the follower robots, thereby maximizing its gains. Additionally, with the escalation of mutual information value, there is a probability that the resource-providing robot opts for decision-making based on others’ increases in information, culminating in a convergence toward the leader–follower model within the game.

The mutual information $I\left(X;Y\right)$ undergoes a mapping through a function to yield a probability value $\lambda_{v}$ within the interval [0, 1]. This mapping is reliant on the characteristics of the exponential function. The exponential function offers a logical method to describe information disparity in probability space, allowing for the representation of a robot’s inclination towards engaging in either the Stackelberg game or the Cournot game.

(6) $$\lambda_{v}\left[I\left(X;Y\right)\right]=1-e^{-I\left(X;Y\right)}$$

The analysis reveals that when $I\left(X;Y\right)=0$, $\lambda_{v}\left[0\right]=1-e^{0}=0$, which implies that in the absence of information exchange, the probability of robots engaging in Stackelberg games is zero, indicating that robots will resort to Cournot games. As $I\left(X;Y\right)$ increases, the function $\lambda_{v}\left[I\left(X;Y\right)\right]$ gradually approaches 1. In this scenario, follower robots tend to trust the strategies of leader robots more. Consequently, leader robots make decisions first, and follower robots react responsively based on the strategies of leader robots. As $I\left(X;Y\right)$ approaches infinity, $\lambda_{v}\left[\infty \right]=1-e^{-\infty }=1$, indicating that the probability of robots engaging in Stackelberg games tends toward 1.

### 3.3. Game Model

In a dynamic scenario, it is assumed that robot $i\left(i=1,2,\cdots V\right)$ can provide homogeneous resources. Each robot’s decision involves selecting the optimal quantity of resources to provide. Decision-making occurs at discrete time periods t=0,1,2,⋯,t, where $x_{i}=x_{i}\left(t\right)$ represents the amount of resources provided by robot i in period t. Thus, the total production of the V robots is denoted by $x=\sum_{i=1}^{V}x_{i}$.

Assuming the variable cost function of the robot takes a linear form, then:

(7) $$C_{i}\left(x_{i}\right)=c_{i}x_{i}$$

Here, $c_{i}$ denotes the total variable cost per unit of product for robot $i\left(i=1,2,\cdots V\right)$. The price *P* at period *t* is determined by the inverse demand function of $P=g\left(x\right)$.

Let the inverse demand function be:

(8) $$P=g\left(x\right)=m-\alpha \sum_{i=1}^{V}x_{i}$$

Here, m represents the intercept of the price, indicating the price when the production volume is zero; α denotes the slope, indicating the rate at which the price decreases with an increase in supply quantity.

Drawing on the definition of utility in decision theory and behavioral economics [34,35], the utility of a robot $i\left(i=1,2,\cdots V\right)$ is defined as the residual utility derived from the total benefits minus the total costs. Then, the utility function is defined as:

(9) $$U_{i}=x_{i}g\left(\sum_{i=1}^{V}x_{i}\right)-c_{i}x_{i}$$

## 4. Model Analysis

In this paper, we will adopt the method of virtual competitors, transforming the multiplayer game of swarm robot systems into a two-player game, assessing the stability of Nash equilibria using the eigenvalues of the Jacobian matrix to theoretically explore the impact of changes in mutual information on the stability of swarm robot systems.

### 4.1. Stability Analysis of the System When $\lambda_{v}\left[I\left(X;Y\right)\right]=0$

From the information model analysis, it is evident that when $\lambda_{v}\left[I\left(X;Y\right)\right]=0$, robots opt for Cournot games among themselves. This implies that robot $i\left(i=1,2\right)$ make independent decisions, and each decision is solely based on their own information without consideration of the strategies of other robots.

In the Cournot game model, each robot decides to choose the quantity of resources to provide to maximize its utility. The simplest method to identify the Nash equilibrium is to compute the first derivative of the utility function for each robot. Hence, the marginal utility for robot $I\left(X;Y\right)$ in the current period is:

(10) $$\frac{\partial U_{i}}{\partial x_{i}}=g\left(\sum_{i=1}^{2}x_{i}\right)+x_{i}\frac{\partial g}{\partial x_{i}}-c_{i}$$

To maximize utility, robots need to make decisions. However, in dynamic scenarios, it is challenging for each robot to acquire complete market information. Therefore, their behavior exhibits adaptability, following a process of bounded rationality based on local estimates established on the previous period’s marginal profits. If a robot estimates that the marginal profit in period t is positive, it will increase output in period t + 1. Conversely, if the marginal profit is negative, it will decrease output. Consequently, the output of robot $i\left(i=1,2\right)$ in period t + 1 can be expressed as:

(11) $$x_{i}\left(t+1\right)=x_{i}\left(t\right)+v_{i}x_{i}\left(t\right)\frac{\partial \Pi_{i}}{\partial x_{i}}$$

In this equation, $v_{i}\left(0<v_{i}<1\right)\left(i=1,2\right)$ stands as a decision parameter, signifying the relative speed of adjustment in the resource-providing quantity of robots.

In this context, the game model can be expressed as:

(12) $$\left\{\begin{array}{l} x_{1}\left(t+1\right)=x_{1}\left(t\right)\left[1+v_{1}\left(m-c_{1}\right)-2\alpha v_{1}x_{1}\left(t\right)-\alpha v_{1}x_{2}\left(t\right)\right]\\ x_{2}\left(t+1\right)=x_{2}\left(t\right)\left[1+v_{2}\left(m-c_{2}\right)-2\alpha v_{2}x_{2}\left(t\right)-\alpha v_{2}x_{1}\left(t\right)\right] \end{array}\right.$$

Let:

(13) $$\left\{\begin{array}{l} x_{1}\left(t\right)\left[1+v_{1}\left(m-c_{1}\right)-2\alpha v_{1}x_{1}\left(t\right)-\alpha v_{1}x_{2}\left(t\right)\right]=x_{1}\left(t\right)\\ x_{2}\left(t\right)\left[1+v_{2}\left(m-c_{2}\right)-2\alpha v_{2}x_{2}\left(t\right)-\alpha v_{2}x_{1}\left(t\right)\right]=x_{2}\left(t\right) \end{array}\right.$$

The four equilibrium points of the system are obtained as: $E_{1}=\left(0,0\right)$, E2=m−c12α,0, E3=0,m−c22α, E4=m+c2−2c13α,m+c1−2c23α. Clearly, $E_{1}$, $E_{2}$ and $E_{3}$ represent bounded equilibria. When $m+c_{2}>2c_{1}$ and $m+c_{1}>2c_{2}$, $E_{4}$ serves as a Nash equilibrium point. Next, we will discuss the conditions under which stable dynamic equilibrium can be achieved in the game model. We will explore whether periodic or chaotic states occur when these parameter conditions for stability are not met. The Jacobian matrix of the system is given by:

(14) $$J=\left(\begin{array}{cc} v_{1}\left(m-c_{1}\right)-4\alpha v_{1}x_{1}-\alpha v_{1}x_{2}+1 & -\alpha v_{1}x_{1}\\ -\alpha v_{2}x_{2} & v_{2}\left(m-c_{2}\right)-4\alpha v_{2}x_{2}-\alpha v_{2}x_{1}+1 \end{array}\right)$$

By analyzing the eigenvalues of J, we can discuss the stability of the Nash equilibrium points of the system. It is evident that points $E_{1}$, $E_{2}$ and $E_{3}$ are unstable.

Next, we analyze the stability of $E_{4}$.

**Theorem** **1.** *When $v_{1}$ and $v_{2}$ satisfy $0<2v_{2}\left(m-2c_{2}+c_{1}\right)-v_{1}v_{2}\left(m-2c_{1}+c_{2}\right)\left(m-2c_{2}+c_{1}\right)+2v_{1}\left(m-2c_{1}+c_{2}\right)+<6$, the Nash equilibrium point is stable.*

**Proof of** **Theorem 1.** The Jacobian matrix at point $E_{4}$ is given by:

(15) $$J=\left(\begin{array}{cc} 1+\frac{2v_{1}\left(2c_{1}-m-c_{2}\right)}{3} & -\frac{v_{1}\left(m+c_{2}-2c_{1}\right)}{3}\\ -\frac{v_{2}\left(m+c_{1}-2c_{2}\right)}{3} & 1+\frac{2v_{2}\left(2c_{2}-m-c_{1}\right)}{3} \end{array}\right)$$

Its characteristic equation is: $\lambda^{2}-trJ\lambda +\det J=0$, where trJ is the trace of J and detJ is the determinant of J.

(16) $$\left\{\begin{array}{c} trJ=2+\frac{2v_{1}\left(2c_{1}-m-c_{2}\right)+2v_{2}\left(2c_{2}-m-c_{1}\right)}{3}\\ \det J=1+\frac{2v_{1}\left(2c_{1}-m-c_{2}\right)+2v_{2}\left(2c_{2}-m-c_{1}\right)}{3}\\ +v_{1}v_{2}\left(2c_{1}-m-c_{2}\right)\left(2c_{2}-m-c_{1}\right) \end{array}\right.$$

We obtain:

(17) $$trJ^{2}-4\det J=4\left\{\begin{array}{c} {\left[\frac{v_{1}\left(2c_{1}-m-c_{2}\right)-v_{2}\left(2c_{2}-m-c_{1}\right)}{3}\right]}^{2}\\ +\frac{v_{1}v_{2}\left(2c_{1}-m-c_{2}\right)\left(2c_{2}-m-c_{1}\right)}{9} \end{array}\right\}$$

As the premise for $E_{4}$ to be a Nash equilibrium point requires $m+c_{2}>2c_{1}$ and $m+c_{1}>2c_{2}$, so $trJ^{2}-4\det J>0$. This indicates that the eigenvalues of Nash equilibrium point $E_{4}$ are real numbers. According to the Jury criterion, the sufficient and necessary condition for the stability of Nash equilibrium point $E_{4}$ is:

(18) $$\left\{\begin{array}{l} 1+trJ+\det J>0\\ 1-trJ+\det J>0\\ \left|\det J\right|<1 \end{array}\right.$$

Solving:

(19) $$0<2v_{1}\left(m+c_{2}-2c_{1}\right)+2v_{2}\left(m+c_{1}-2c_{2}\right)-v_{1}v_{2}\left(m+c_{2}-2c_{1}\right)\left(m+c_{1}-2c_{2}\right)<6$$

Clearly, the Nash equilibrium point $E_{4}$ is a stable equilibrium point within the parameter range defined by Equation (19). However, if the parameter exceeds this range, the equilibrium point will become unstable. The stability of the system at the Nash equilibrium point $E_{4}$ depends on the system parameter, influenced by each parameter in Equation (19).  □

### 4.2. Stability Analysis of the System When $\lambda_{v}\left[I\left(X;Y\right)\right]\to 1$

If $\lambda_{v}\left[I\left(X;Y\right)\right]\to 1$, robots opt for Stackelberg games among themselves. In this scenario, the robot acting as the leader prioritizes decision-making, while follower robots reactively make decisions based on the strategies of the leader robot.

Similarly, in the leader–follower model, referring to Equation (10), the first-order boundary condition is obtained as follows:

(20) $$x_{i}=\frac{m-c_{i}-\alpha x_{j}}{2\alpha }$$

In the game model, let robot 2 be the leader robot, capable of foreseeing the output of robot 1 and the relevant information. Furthermore, when anticipating future economic variables, robot 2 not only considers their previous expectations of these variables but also adjusts them using past forecast errors. Therefore, the output of robot 2 in period t + 1 takes the following form:

(21) $$x_{2}\left(t+1\right)=x_{2}\left(t\right)+v_{2}\left(x_{2}\left(t\right)-{x^{*}}_{2}\left(t\right)\right)$$

where $v_{2}\left(0<v_{2}<1\right)$ is the adaptation coefficient, i.e., the correction coefficient.

In this case, we obtain the following game model:

(22) x1t+1=x1t1+v1m−c1−2αβx1t−αβx2tx2t+1=1−v2x2t+v22αm−c2−αx1t

Let:

(23) x1t1+v1m−c1−2αβx1t−αβx2t=x1t1−v2x2t+v22αm−c2−αx1t=x2t

The system’s two equilibrium points can be derived as: E1=0,m−c22α and E2=m+c2−2c13α,m+c1−2c23α. Clearly, $E_{1}$ represents a bounded equilibrium. When $m+c_{2}>2c_{1}$ and $m+c_{1}>2c_{2}$, $E_{2}$ serves as the Nash equilibrium point. Similarly, by calculating the Jacobian matrix of the system, we can investigate the local stability of equilibrium points and local bifurcation behavior.

(24) J=1+v1m−4αv2x1−αx2−c1−αv1x1−v221−v2

By computing the eigenvalues of J, it is evident that J is an unstable point.

Next, we analyze the stability of $E_{2}$.

**Theorem** **2.** When $v_{1}$ and $v_{2}$ satisfy $2v_{2}+4v_{1}\alpha x_{1}^{*}-\frac{3}{2}v_{2}v_{1}\alpha x_{1}^{*}-4<0$ and $\frac{3}{2}v_{2}v_{1}\alpha x_{1}^{*}-v_{2}-2v_{1}\alpha x_{1}^{*}<0$, the Nash equilibrium point $E_{2}$ is stable. Where $x_{1}^{*}=\frac{m+c_{2}-2c_{1}}{3\alpha }$, $x_{2}^{*}=\frac{m+c_{1}-2c_{2}}{3\alpha }$.

**Proof of** **Theorem 2.** The Jacobian matrix at point $E_{2}$ is given by:

(25) $$J=\left(\begin{array}{cc} 1-2v_{1}\alpha x_{1}^{*} & -v_{1}\alpha x_{1}^{*}\\ -\frac{v_{2}}{2} & 1-v_{2} \end{array}\right)$$

Similarly, the characteristic equation is: $\lambda^{2}-trJ\lambda +\det J=0$. Thus, we obtain:

(26) $$\left\{\begin{array}{l} trJ=2-v_{2}-2v_{1}\alpha x_{1}^{*}\\ \det J=1-v_{2}-2v_{1}\alpha x_{1}^{*}+\frac{3}{2}v_{1}v_{2}\alpha x_{1}^{*} \end{array}\right.$$

Computing:

(27) $$trJ^{2}-4\det J=4{\left(v_{2}-2v_{1}\alpha x_{1}^{*}\right)}^{2}+2v_{1}v_{2}\alpha x_{1}^{*}>0$$

Similarly, according to the Jury criterion, the sufficient and necessary condition for the stability of Nash equilibrium point $E_{2}$ can be derived as:

(28) $$\left\{\begin{array}{l} 2v_{2}+4v_{1}\alpha x_{1}^{*}-\frac{3}{2}v_{1}v_{2}\alpha x_{1}^{*}-4<0\\ \frac{3}{2}v_{1}v_{2}\alpha x_{1}^{*}-v_{2}-2v_{1}\alpha x_{1}^{*}<0 \end{array}\right.$$

This indicates that the Nash equilibrium point $E_{2}$ is a stable equilibrium point within the parameter range defined by Equation (28).  □

### 4.3. Stability Analysis of the System When $0<\lambda_{v}\left[I\left(X;Y\right)\right]<1$

When $\lambda_{v}\left[I\left(X;Y\right)\right]$ takes values between 0 and 1, the weighting of the Cournot and Stackelberg game models can be adjusted through an interpolation function, enabling the model to operate reasonably under different levels of trust. In this paper, decisions under both scenarios are weighted according to the proportion of $\lambda_{v}\left[I\left(X;Y\right)\right]$. Therefore, the model is defined as:

(29) $$\left\{\begin{array}{l} x_{1}\left(t+1\right)=\left(1-\lambda_{v}\left[I\left(X;Y\right)\right]\right)x_{1}^{Cournot}\left(t\right)+\lambda_{v}\left[I\left(X;Y\right)\right]x_{1}^{Stackelberg}\left(t\right)\\ x_{2}\left(t+1\right)=\left(1-\lambda_{v}\left[I\left(X;Y\right)\right]\right)x_{2}^{Cournot}\left(t\right)+\lambda_{v}\left[I\left(X;Y\right)\right]x_{2}^{Stackelberg}\left(t\right) \end{array}\right.$$

## 5. Simulation Analysis

In this section, MATLAB R2018b is employed to simulate the dynamic complexity of the swarm robots’ game model to better observe the dynamic complexity features exhibited when parameters are outside the stable domain, focusing on varying levels of mutual information.

### 5.1. Parameter Settings

Parameter settings: m = 8, α = 1.5, $c_{1}$ = 1.5, $c_{2}$ = 1.2, $v_{2}=0.35$. Through dynamic system modeling and simulation using software, we can observe the decision complexity of robots under different levels of mutual information.

### 5.2. Simulation

#### 5.2.1. Simulation Analysis When $\lambda_{v}\left[I\left(X;Y\right)\right]=0$

**Figure 2:**
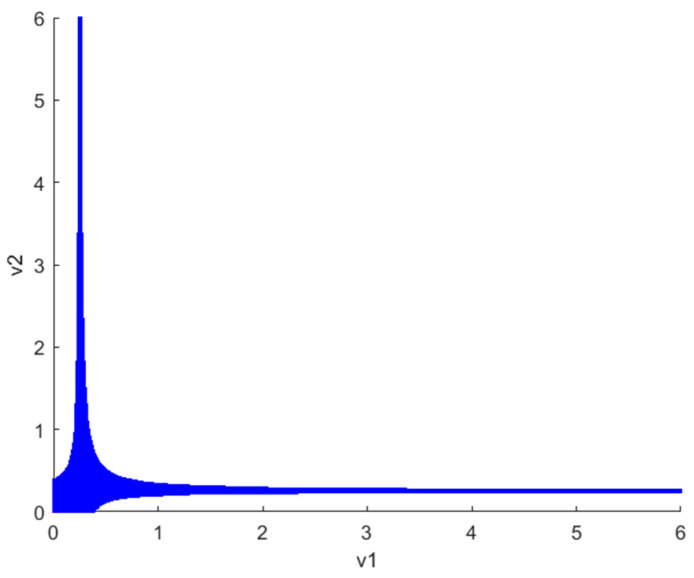
The stability region plot of Nash equilibrium points $E_{4}$ when $\lambda_{v}\left[I\left(X;Y\right)\right]=0$ is illustrated.

Figure 2 illustrates the stability region plot of $v_{1}$ and $v_{2}$ at the Nash equilibrium when $\lambda_{v}\left[I\left(X;Y\right)\right]=0$. It can be observed that when the decision parameters ($v_{1}$ and $v_{2}$) of robots 1 and 2 vary within the shaded region, the Nash equilibrium is stable. Notably, $v_{1}$ and $v_{2}$ decrease independently as the other increases, indicating that both robots are inclined towards adopting competitive strategies. In addition, the figure illustrates the symmetry of the stabilization regions for $v_{1}$ and $v_{2}$ under the Nash equilibrium. This symmetry suggests that the chaotic behavior exhibited by the system remains consistent when $v_{1}$ and $v_{2}$ are varied within a specified range. Consequently, the subsequent discussion will concentrate on the decision parameter $v_{1}$ and investigate its impact on the system’s stability and chaotic behavior.

**Figure 3:**
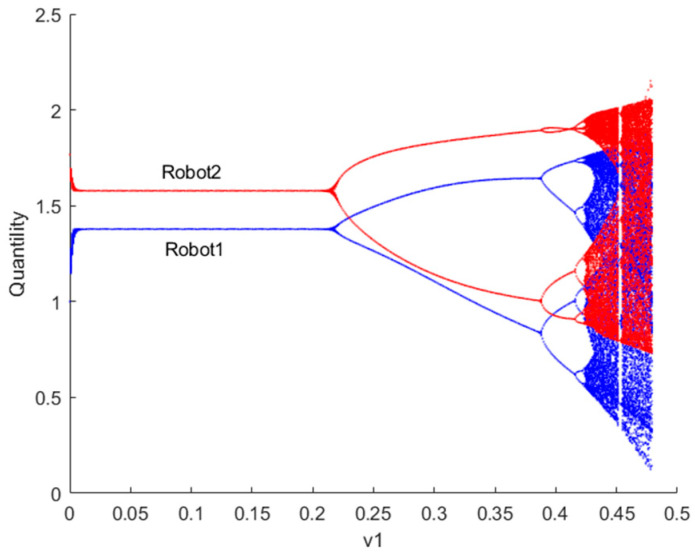
Bifurcation diagram of the quantity of resources to provide for robots 1 and 2 when $\lambda_{v}\left[I\left(X;Y\right)\right]=0$.

Figure 3 illustrates the bifurcation diagram of the quantity of resources to provide for robots 1 and 2 as the decision parameter $v_{1}$ changes when $\lambda_{v}\left[I\left(X;Y\right)\right]=0$. If $\lambda_{v}\left[I\left(X;Y\right)\right]=0$, the robot $i\left(i=1,2\right)$ engages in Cournot games. From the graph, it can be observed that when the decision parameter $v_{1}$ > 0.213, the Nash equilibrium point loses stability. Contrary to common belief, higher output does not necessarily lead to greater utility. In the game process, the variation in robots’ adjustment speed of the quantity of resources to provide in the game market leads to complex dynamics in utility. If the current utility of robots is positive, increasing the quantity of resources to provide adjustment speed in the next period can gain more advantages. However, when the adjustment speed exceeds a certain value, the system transitions from Cournot game equilibrium to a chaotic state. In chaotic states, the decision-making of robots no longer exhibits a singular optimization pattern but rather demonstrates complex dynamic behaviors.

**Figure 4:**
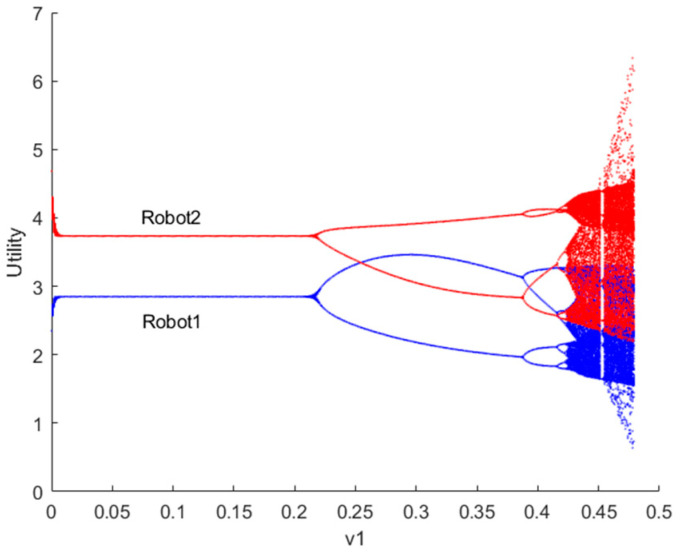
Bifurcation diagram of utility for robots 1 and 2 when $\lambda_{v}\left[I\left(X;Y\right)\right]=0$.

Figure 4 depicts the bifurcation diagram of utility for robots 1 and 2 as the decision parameter $v_{1}$ changes when $\lambda_{v}\left[I\left(X;Y\right)\right]=0$. It is evident that the smaller the adjustment speed $v_{1}$ of resource provision for robots, the more stable their utility, and vice versa. This observation aligns with reality, as slower reaction speeds of robots to the market correspond to slower adjustments in resource provision, leading to more stable utility for both the market and the robots. When the system falls into a chaotic state, robots will struggle to make long-term strategic plans and unlikely achieve stable utility. Additionally, due to significant fluctuations in the game market, robots’ resource provision adjustment speeds will struggle to keep up with market changes, leading to situations of oversupply (resource backlog) or excess supply (being occupied by rival robots), which is undesirable for both parties.

#### 5.2.2. Simulation Analysis When $\lambda_{v}\left[I\left(X;Y\right)\right]\to 1$

**Figure 5:**
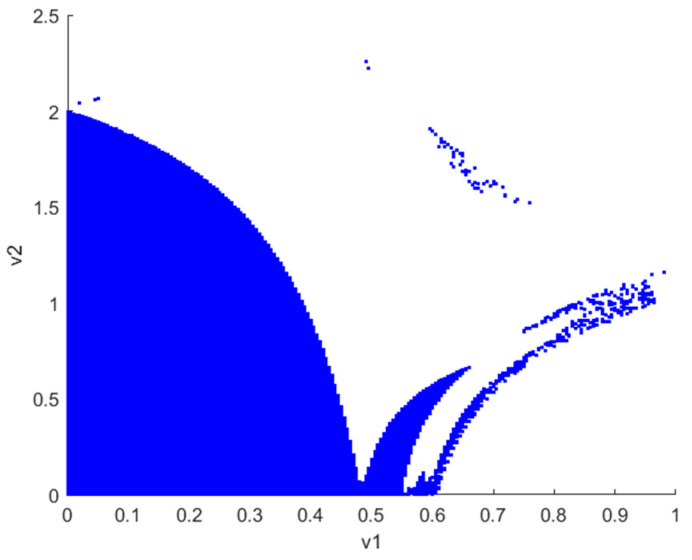
The stability region plot of Nash equilibrium points $E_{2}$ when $\lambda_{v}\left[I\left(X;Y\right)\right]\to 1$ is illustrated.

Figure 5 illustrates the stability region plot of $v_{1}$ and $v_{2}$ at the Nash equilibrium when $\lambda_{v}\left[I\left(X;Y\right)\right]\to 1$. It can be observed that when the decision parameters ($v_{1}$ and $v_{2}$) of robots 1 and 2 vary within the shaded region, the Nash equilibrium is stable. With the increase in $v_{1}$, the swarm robot system will enter a chaotic state. Notably, the decision parameter $v_{2}$ can take any value within its defined domain, i.e., ($0<v_{2}<1$). In contrast, the decision parameter $v_{1}$ has a certain upper limit ($v_{1}>1$), beyond which the neighborhood of the equilibrium becomes unstable or even chaotic, leading to bifurcation. Thus, $v_{1}$ is the primary parameter driving the system towards chaos.

**Figure 6:**
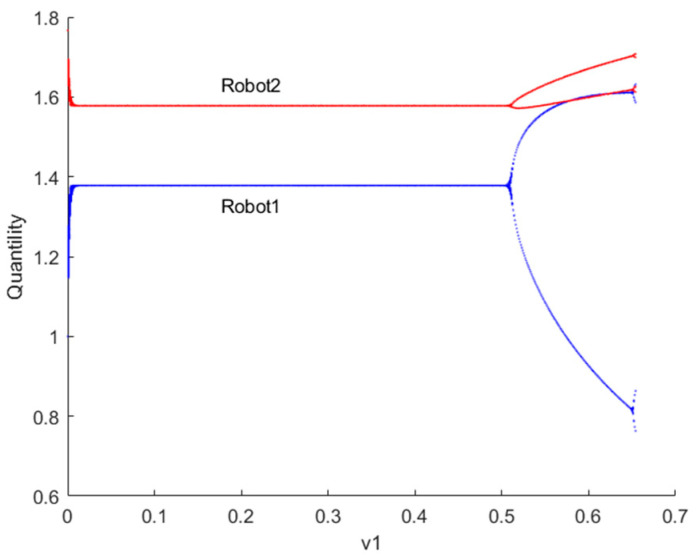
Bifurcation diagram of the quantity of resources to provide for robots 1 and 2 when $\lambda_{v}\left[I\left(X;Y\right)\right]\to 1$.

Figure 6 illustrates the bifurcation diagram of resource provision for robots 1 and 2 as the decision parameter $v_{1}$ changes when $\lambda_{v}\left[I\left(X;Y\right)\right]\to 1$. If $\lambda_{v}\left[I\left(X;Y\right)\right]\to 1$, robots engage in Stackelberg games. It can be observed that the output of robots 1 and 2 fluctuates near the predicted equilibrium point. If the current profit of robots is positive, increasing the output adjustment speed in the next period can gain more advantage. When the adjustment speed exceeds $v_{1}$ = 0.505, the system transitions from a Stackelberg game-predicted equilibrium to a chaotic state.

**Figure 7:**
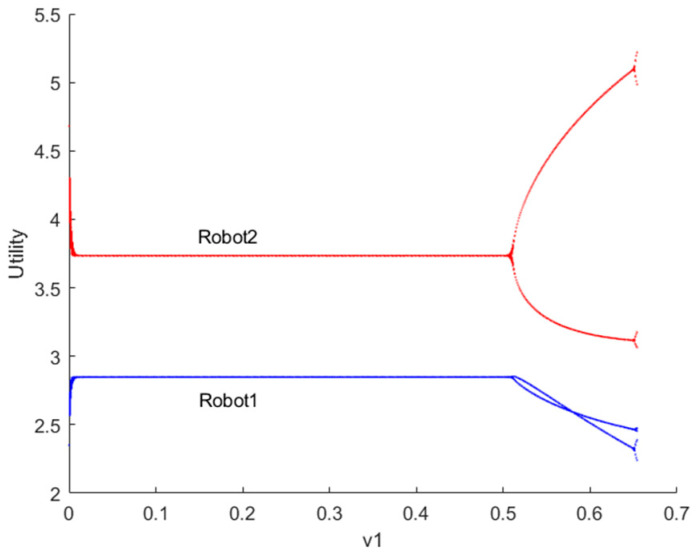
Bifurcation diagram of utility for robots 1 and 2 when $\lambda_{v}\left[I\left(X;Y\right)\right]\to 1$.

Figure 7 illustrates the bifurcation diagram of utility for robots 1 and 2 as the decision parameter $v_{1}$ changes when $\lambda_{v}\left[I\left(X;Y\right)\right]\to 1$. From the graph, it is evident that the equilibrium utility curves for both robots are horizontal lines, indicating that the equilibrium utility does not vary with the adjustment speed of the output. Since robot 2 acts as the output leader in the Stackelberg model, it possesses slightly higher equilibrium utility compared to robot 1. In pursuit of greater profits, robot 2 continuously increases the output adjustment speed $v_{1}$, which consequently impacts the utility of robot 1. As a result, the equilibrium utility becomes unstable, and the system enters a state of bifurcation and chaos.

#### 5.2.3. Simulation Analysis When $0<\lambda_{v}\left[I\left(X;Y\right)\right]<1$

**Figure 8:**
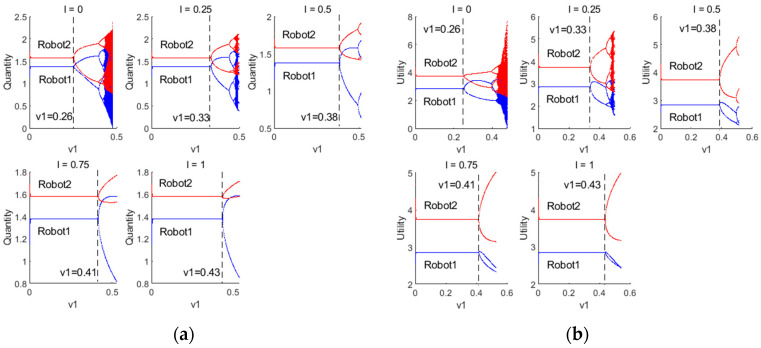
(**a**) Bifurcation diagram of the quantity of resources to provide for robots 1 and 2 under different $I\left(X;Y\right)$; (**b**) Bifurcation diagram of the utility for robots 1 and 2 under different $I\left(X;Y\right)$.

Figure 8 illustrate the bifurcation diagrams of resource provision and utility for robots 1 and 2 under different $I\left(X;Y\right)$ values as the decision parameter $v_{1}$ changes. It can be observed that as the level of information interaction between robots increases (i.e., as $I\left(X;Y\right)$ increases), the decision-making pattern of robots gradually transitions from Cournot to Stackelberg games. Simultaneously, the sensitivity of robots 1 and 2 to changes in the decision parameter decreases. This decrease in sensitivity leads to a reduced probability of entering a chaotic state compared to when $I\left(X;Y\right)=0$. When $I\left(X;Y\right)=0$, robots 1 and 2 simultaneously determine their decision without interfering with each other, resulting in a complex dynamic system. As $I\left(X;Y\right)$ increases, the increased frequency and effectiveness of information interaction among robots facilitate more comprehensive information flow and sharing within the system. In this scenario, robot 2 acts as the leader, making decisions first, while robot 1 responds after the leader’s quantity of resources to provide is determined. This leader–follower structure enables robot 2 to better anticipate the behavior of robot 1 and adjust its strategy accordingly. As a result, the sensitivity to changes in the decision parameter $v_{1}$ decreases, leading to a more stable system and reducing the likelihood of entering a chaotic state.

#### 5.2.4. Fractal Phenomenon Simulation Analysis

**Figure 9:**
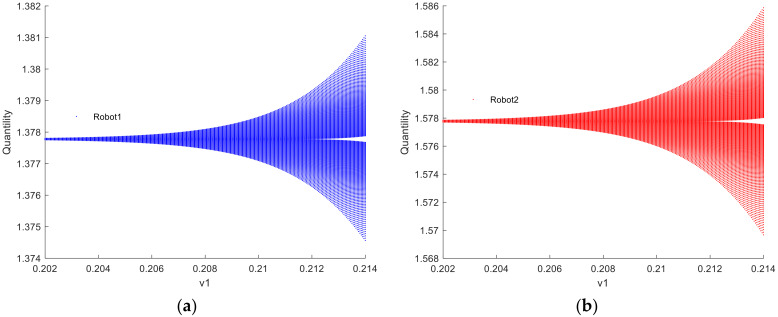
(**a**,**b**) Fractal phenomenon of robots 1 and 2 when $\lambda_{v}\left[I\left(X;Y\right)\right]=0$.

**Figure 10:**
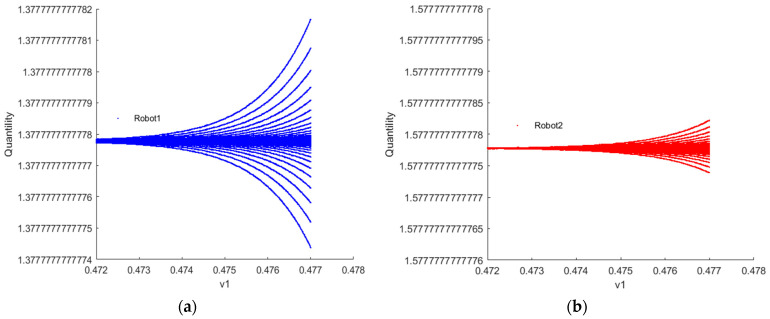
(**a**,**b**) Fractal phenomenon of robots 1 and 2 when $\lambda_{v}\left[I\left(X;Y\right)\right]\to 1$.

Figure 9 and Figure 10 depict the fractal phenomenon observed when $\lambda_{v}\left[I\left(X;Y\right)\right]=0$ and $\lambda_{v}\left[I\left(X;Y\right)\right]\to 1$, respectively, indicating the chaotic state of robots 1 and 2. This occurrence can be interpreted as a result of the complexity and nonlinearity arising from internal interactions within the system. Upon magnification by a factor of 10,000, fractal patterns are observed in the bifurcation diagrams of the quantity of resources to provide for robots 1 and 2. This observation suggests that the behavior of the robotic swarm system exhibits extremely intricate and seemingly disorderly yet actually structured temporal dynamics. The scale invariance of this phenomenon implies that the system’s behavior retains similar characteristics regardless of whether observed at the magnified scale or the original scale. The emergence of fractal patterns implies the dynamic stability of the system. Even amidst bifurcations and complex variations, the system maintains a degree of stability, preserving its structural and behavioral features within a certain range. This suggests that the bifurcation critical point is not a fixed point but rather a critical point of the system in different states, maintaining a certain level of stability within a defined range.

## 6. Conclusions

This paper, set against the backdrop of the division of labor in swarm robotics systems, applies information theory, game theory, and the bifurcation and chaos theory of nonlinear dynamics to analyze, theoretically, the impact of the information interaction among individual robots on the stability of the system. To begin with, we construct a mutual information model to depict the interactions of information between individuals by leveraging mutual information, treating each robot as a random variable with its behavior and decisions as observed values. The transformation of mutual information into probability is described through the utilization of an exponential function. Subsequently, this transformed probability is employed to analyze the impact of the disparity of mutual information on the decision-making behaviors exhibited by individual robots. Furthermore, a game model for swarm robotics systems is established to explore the complex dynamic behaviors exhibited by swarm robots under disparity mutual information during the game process. The focus is on analyzing the decision-making of robots and the system’s stability. It is pointed out how swarm robots select appropriate parameter values for decision-making based on the stability range of each parameter at Nash equilibrium points. Lastly, dynamic complexity simulations of the game models are simulated under the disparity mutual information values.

By introducing the concept of mutual information into the division of labor decision-making problem of swarm robotics, an economic perspective is employed to analyze the system stability and complex dynamics of swarm robots under the disparity of mutual information. The simulation results indicate that as the mutual information $I\left(X;Y\right)$ increases, the decision-making pattern of the swarm robots shifts gradually from the Cournot competition to the Stackelberg competition. Additionally, the sensitivity of robots 1 and 2 to changes in decision parameter $v_{1}$ decreases. Compared to the scenario where $I\left(X;Y\right)$, the probability of the swarm robot system entering a chaotic state also decreases. This underscores the importance of information interaction in the decision-making process of swarm robots. By utilizing mutual information, we can better comprehend the information interaction among robots and adjust decision strategies accordingly, thereby enhancing the stability of the system.

Next, the authors plan to delve deeper into the role of mutual information in robot swarms. Specifically, we aim to utilize mutual information to assess the level of collaboration and cooperation among robots and explore the integration of chaos control to optimize the cooperative behavior of the swarm robots. Chaos control can assist in regulating the system’s dynamic behavior, making it more stable or adaptable to specific task requirements. By analyzing mutual information, the author will guide the implementation of chaos control to ensure that the robot swarm can maintain stability or flexibility in dynamic environments, thereby enhancing the overall performance and efficiency of the robot team. Additionally, the virtual competitor approach employed in this study, which transforms a multi-player game model into a two-player game, has certain limitations. To address these limitations, future research will expand to include group dynamic simulations with more than two robots. This extension will enhance the realism of the simulation results and offer more effective solutions to practical problems.

## Data Availability

Data are contained within the article.
